# A State-of-the-Art Review of EEG-Based Imagined Speech Decoding

**DOI:** 10.3389/fnhum.2022.867281

**Published:** 2022-04-26

**Authors:** Diego Lopez-Bernal, David Balderas, Pedro Ponce, Arturo Molina

**Affiliations:** Tecnologico de Monterrey, National Department of Research, Mexico City, Mexico

**Keywords:** EEG, BCI, review, imagined speech, artificial intelligence

## Abstract

Currently, the most used method to measure brain activity under a non-invasive procedure is the electroencephalogram (EEG). This is because of its high temporal resolution, ease of use, and safety. These signals can be used under a Brain Computer Interface (BCI) framework, which can be implemented to provide a new communication channel to people that are unable to speak due to motor disabilities or other neurological diseases. Nevertheless, EEG-based BCI systems have presented challenges to be implemented in real life situations for imagined speech recognition due to the difficulty to interpret EEG signals because of their low signal-to-noise ratio (SNR). As consequence, in order to help the researcher make a wise decision when approaching this problem, we offer a review article that sums the main findings of the most relevant studies on this subject since 2009. This review focuses mainly on the pre-processing, feature extraction, and classification techniques used by several authors, as well as the target vocabulary. Furthermore, we propose ideas that may be useful for future work in order to achieve a practical application of EEG-based BCI systems toward imagined speech decoding.

## 1. Introduction

One of the main technological objectives in our current era is to generate a connected environment in which humans can be able to create a link between their daily and real life physical activities and the virtual world (Chopra et al., 2019). This type of applications are currently developed under a framework denominated as Future Internet (FI). There is a wide range of technological implementations that can benefit from FI, such as human-computer interaction and usability (Haji et al., 2020). For example, speech driven applications such as Siri and Google Voice Search are widely used in our daily life to interact with electronic devices (Herff and Schultz, 2016). These applications are based on a speech recognition algorithm, which allows the device to convert human voice to text. Nevertheless, there are certain health issues that may impede some people from using these applications.

Verbal communication loss can be caused by injuries and neurodegenerative diseases that affect the motor production, speech articulation, and language understanding. Few examples of these health issues include stroke, trauma, and amyotrophic lateral sclerosis (ALS) (Branco et al., 2021). In some cases, these neurodegenerative conditions may lead patients to fall into a locked-in syndrome (LIS), in which they are not capable to communicate due to the complete loss of motor control.

To address this problem, Brain Computer Interfaces (BCI) have been proposed as an assistive technology to provide a new communication channel for those individuals with LIS. BCI technologies offer a bridge between the brain and outer world, in such a way that it creates a bi-directional communication interface which reads the signals generated by the human brain and converts them into the desired cognitive task (Gu et al., 2021; Rasheed, 2021; Torres-Garćıa et al., 2022). In such manner, a thought-to-speech interface can be implemented so that people who are not able to speak due to motor disabilities can use their brain signals to communicate without the need of moving any body part.

Generally speaking, BCI for imagined speech recognition can be decomposed into four steps:

1. Signal acquisition: this step involves a deep understanding of the properties of the signals that are being recorded, as well as how the signals are going to be captured.
2. Pre-processing: the main objective of this step is to unmask and enhance the information and patterns within the signal.
3. Feature extraction: this step involves the extraction of the main characteristics of the signal.
4. Classification: this is the final step, in the different mental states are classified depending on their features.

Several methods, both invasive and non-invasive, have been proposed and studied in order to acquire the signals that the brain produce during the speech imagining process. Some of these methods are magnetoencephalography (MEG), functional magnetic resonance imaging (fMRI), functional near-infrared spectroscopy (fNIRS), electrocardiography (ECOG), and electroencelography (EEG) (Sereshkeh et al., 2018; Angrick et al., 2019; Dash et al., 2020b; Fonken et al., 2020; Si et al., 2021). Invasive methods, such as ECOG, have proven to provide, in average, greater classifying accuracies than non-invasive methods (MEG, fMRI, fNIRS, and EEG) during imagined speech decoding. In fact, invasive techniques have more easily exceeded the threshold for practical BCI imagined speech application (70%), in contrast to non-invasive techniques (Sereshkeh et al., 2018). Among the mentioned techniques for imagined speech recognition, EEG is the most commonly accepted method due to its high temporal resolution, low cost, safety, and portability (Saminu et al., 2021). Nevertheless, speech-based BCI systems using EEG are still in their infancy due to several challenges they have presented in order to be applied to solve real life problems.

One of the main challenges that imagined speech EEG signals present is their low signal-to-noise ratio (SNR). This low SNR cause the component of interest of the signal to be difficult to recognize from the background brain activity given by muscle or organs activity, eye movements, or blinks. Furthermore, even EEG equipment is sensitive enough to capture electrical line noise from the surroundings (Bozhkov and Georgieva, 2018). Moreover, despite EEG having high temporal resolution, it lacks from spatial resolution which can lead to low accuracy on the source of information on the brain cortex, distortion of topographical maps by removing high spatial frequency, and difficulty to reject artifacts from the main signal (Kwon et al., 2019). Because of these issues, classical machine learning (ML) methods that have proven to be successful in the recognition of motor imagery tasks have not obtained good performance when applied to imagined speech recognition. Thus, deep learning (DL) algorithms, along with various filtering and feature extraction techniques, have been proposed to enhance the performance of EEG-based BCI systems (Antoniades et al., 2016).

That being said, imagined speech recognition has proven to be a difficult task to achieve within an acceptable range of classification accuracy. Therefore, in order to help researchers to take the best decisions when approaching this problem, the main objective of the present review is to provide an insight about the basics behind EEG-based BCI systems and the most recent research about their application toward imagined speech decoding, as well as the most relevant findings on this area. The rest of the paper is organized as follows: Section 2 investigates the current applications of BCI systems and their classification. Section 3 discusses the characteristics of electroencephalography (EEG) and the different frequency bands that can be found in it. Section 4 presents the different prompts that have been studied in literature; while Sections 5, 6, and 7 discuss about the pre-processing, feature extraction and classification techniques, respectively. Section 8 offers a summary of the reviewed works and techniques. Finally, Section 9 presents the findings of this work and proposes future directions for the improvement of imagined speech recognition.

## 2. Brain Computer Interface

**Figure 1:**
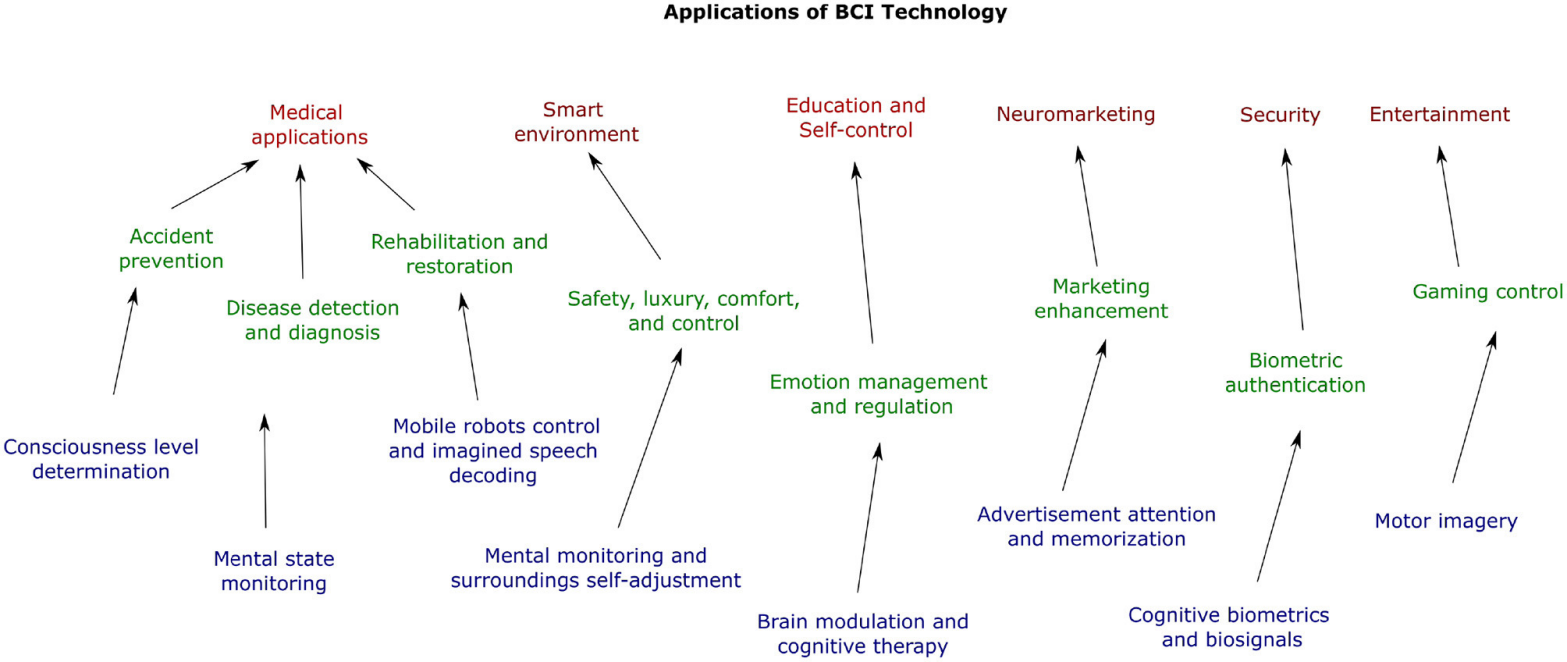
Technology map of BCI applications.

The advent of Future Internet has caused a widespread connectivity between everyday electronic devices and the human body (Zhang et al., 2018). One example is Brain Computer Interface, which is a technology that uses brain activity and signals to create a communication channel between external electronic devices and the human brain (Abiri et al., 2019). BCI has been used for several applications in various areas, as shown in Figure 1. For example, BCI systems have been applied toward neuromarketing, security, entertainment, smart-environment control, emotional education, among others (Abdulkader et al., 2015; Abo-Zahhad et al., 2015; Aricò et al., 2018; Padfield et al., 2019; Mudgal et al., 2020; Suhaimi et al., 2020; Moctezuma and Molinas, 2022). One of the most explored applications of BCI is toward the medical area to treat and diagnose neurological disorders such as epilepsy, depression, dementia, Alzheimer's, brain stroke, among others (Subasi, 2007; Morooka et al., 2018; Saad Zaghloul and Bayoumi, 2019; Hashimoto et al., 2020; Rajagopal et al., 2020; Sani et al., 2021). Moreover, it has also been used to recognize and classify emotions (Kaur et al., 2018; Suhaimi et al., 2020) and sleep stages (Chen et al., 2018), as well as to bring the opportunity of performing normal movements to people with motor disabilities (Antelis et al., 2018; Attallah et al., 2020; Al-Saegh et al., 2021; Mattioli et al., 2022). Furthermore, one of the most interesting, yet difficult, tasks that are being tried to be accomplished using BCI is imagined speech recognition, in which the objective is to convert the input brain signal to text, sound, or control commands. Different types of BCI systems have been proposed by researchers to be able to use them in real-life scenarios. Some of the most important BCI classifications are: synchronous vs. asynchronous, online vs. offline, exogenous vs. endogenous, invasive vs. non-invasive (Portillo-Lara et al., 2021).

Synchronous BCI are systems that cannot be used freely by the users because they are fixed in determined periods of time. This means that, for imagined speech decoding, the user needs a cue that indicates when to begin the imagination process. Then, the selected time window is analyzed, discarding any other EEG signals that do not belong to that time constraint. On the other hand, asynchronous BCI can be used without any time constraint and they do not need any cue, meaning that it is a more natural process that can be more practical toward real-life applications. However, these systems have shown less accuracy than synchronous ones because of the difficulty on distinguishing intentional mental activity from unintentional one (Han et al., 2020).

Among BCI classification, there are also online and offline systems. Online BCI, just as asynchronous BCI, are promising toward real-life applications because they allow real-time data processing. In other words, during an online setting, the feature extraction and classification processes are done several times during each trial. However, because of this same advantage, the computational complexity that an online system can employ is limited. On the other hand, offline systems do not have this problem as they can use as much computational resources as needed because the feature extraction and classification processes are done until all trails are available and the sessions are over. Nevertheless, because of this same reason, an offline BCI system will be hardly applied under real-life circumstances (Chevallier et al., 2018).

Depending of the type of stimulus that the BCI uses, there can be exogenous and endogenous systems. Exogenous ones use external stimulus to generate the desired neural activation; while endogenous ones can operate independently of any stimulus. For a real-life application of imagined speech decoding, the most appropriate between these two systems would be the endogenous BCI (Lee et al., 2021a).

Brain computer interfaces can also be classified as invasive and non-invasive. The invasive techniques, despite offering the best representation of the brain signals, have the risk of scaring brain tissue, at the same time that are more costly and difficult to use. On the other hand, non-invasive techniques, such as EEG, are used through scanning sensors or electrodes fixed on the scalp to record the brain signals. Due to its easiness to use, its portability and its safety, EEG based BCI have been broadly explored to be applied toward imagined speech recognition.

## 3. Electroencephalography (EEG)

Electroencephalography, also known as EEG, is the most common non-invasive method to measure the electrical activity of the human brain. The signals are acquired by electrodes placed over the scalp that record the voltage difference generated during neural communication (Singh and Gumaste, 2021). The electrodes are then connected to an amplifier and are typically distributed in a standard 10–20 placement (Sazgar and Young, 2019). Commonly, EEG systems consist of 14–64 electrodes (also called channels), thus creating a multi-dimensional signal.

Along with its easiness to use and safety, EEG also has a high temporal resolution, characteristics that make it the most suitable option for imagined speech recognition. The reason behind this is that the analysis of imagined speech signals requires to track how the signal changes over time. However, one of the main disadvantages of EEG is that it can be easily contaminated by surrounding noise caused by external electronic devices. Hence, before being able to analyze EEG waves for imagined speech tasks, they must be pre-processed to enhance the most important information within the signal.

### 3.1. EEG Waves

EEG waves consist of a mixture of diverse base frequencies. These frequencies have been arranged on five different frequency bands: gamma (>35 Hz), beta (12–35 Hz), alpha (8–12 Hz), theta (4–8 Hz), and delta (0.5–4 Hz) (Abhang et al., 2016). Each frequency band represents a determined cognitive state of the brain. Each of these frequency bands plays a determined role at the different stages of speech processing. Thus, recognizing them may aid to better analyze the EEG signal.

- **Gamma waves**. Changes in high gamma frequency (70–150 Hz) are associated with overt and covert speech. According to Pei et al. (2011), during overt speech the temporal lobe, Broca's area, Wernicke's area, premotor cortex and primary motor cortex present high gamma changes. On the other hand, this study also presents evidence of high gamma changes during covert speech in the supramarginal gyrus and superior temporal lobe.
- **Beta waves**. These waves are often related with muscle movement and feedback. Therefore, it can be considered that they are involved during auditory tasks and speech production (Bowers et al., 2013).
- **Alpha waves**. During language processing, these waves are involved in auditory feedback and speech perception. Moreover, alpha frequency during covert speech has been identified as weak in comparison to its behavior during overt speech (Jenson et al., 2014).
- **Theta waves**. According to Kösem and Van Wassenhove (2017), these waves become active during the phonemic restoration, and processing of co-articulation cues to compose words. Also, another study (Ten Oever and Sack, 2015), identified that theta waves can help to identify consonants in syllables.
- **Delta waves**. Intonation and rhythm during speech perception have been found to fall into frequency ranges that belong to the lower delta oscillation band (Schroeder et al., 2008). Also, diverse studies have found other speech processes in which delta waves are involved, such as prosodic phrasing, syllable structure, long syllables, among others (Peelle et al., 2013; Ghitza, 2017; Molinaro and Lizarazu, 2018; Boucher et al., 2019).

## 4. Imagined Speech Prompts in Literature

As said in Section 2, the main objective of applying BCI toward imagined speech decoding is to offer a new communication channel to people who are not able to speak due to any given motor disability. However, as language can be decomposed in several parts, as syllables, phonemes, vocals, and words, several studies have been carried on in order to classify these different parts of language.

In D'Zmura et al. (2009), Brigham and Kumar (2010), and Deng et al. (2010), volunteers imagined two syllables, /ba/ and /ku/. For these studies, the volunteers were given an auditory cue indicating the syllable to be imagined. Another study done by Callan et al. (2000) focused on the imagined speech process of /a/, /i/, and /u/ vowels during a metal rehearsing process after speaking them out loud. DaSalla et al. (2009) also studied /a/, and /u/ vowels using a visual cue for both of them. Those vowels were chosen because of them causing similar muscle activation during real speech production. Also, in a study done by Zhao and Rudzicz (2015) seven phonetic/syllabic prompts were classified during a covert speech production process. In more recent works (Jahangiri et al., 2018, 2019) four phonemic structures (/ba/, /fo/, /le/, and /ry/) were analyzed. The difference between these studies was that in Jahangiri et al. (2018) they used a visual cue, while in Jahangiri et al. (2019) it was an auditory one. Some other studies such as Cooney et al. (2019), Tamm et al. (2020), and Ghane and Hossain (2020) have analyzed EEG signals produced during the imagined speech process of five vowels: /a/, /e/, /i/, /o/, and /u/. Besides phonemes, vowels, and syllables, there have been other studies that have worked with imagined words. For example, Wang et al. (2013) studied the classification of two imagined Chinese characters, whose meanings were “left” and “one.” In González-Castañeda et al. (2017), a study was done to classify five different imagined words: “up,” “down,” “left,” “right,” and “select.” Very similarly, the work done in Pawar and Dhage (2020) worked over the same prompts, with exception of the word “select.” Also, in the study done by Mohanchandra and Saha (2016), they used as prompts five words, being them, namely “water,” “help,” “thanks,” “food,” and “stop.” In Zhao and Rudzicz (2015), apart from the phonetic classification, they also worked toward the classification of the imagined words “pat,” “pot,” “knew,” and “gnaw”; where “pat”/“pot” and “knew”/“gnaw” are phonetically similar. Furthermore, in Nguyen et al. (2017) two different groups of imagined words (short and long) were analyzed. The former consisted on the words “in,” “out,” and “up,” while the latter consisted on “cooperate” and “independent.”

## 5. Pre-processing Techniques in Literature

As mentioned previously, EEG signals can be easily contaminated by external noise coming from electrical devices and artifacts such as eye blinks, breathing, etc. In order to diminish the noise and increase the SNR of the EEG waves, several pre-process techniques have been proposed in literature. Moreover, pre-processing is important because it can help to reduce the computational complexity of the problem and, therefore, to improve the efficiency of the classifier (Saminu et al., 2021). Generally speaking, pre-processing of EEG signals is usually formed by downsampling, band-pass, filtering, and widowing (Roy et al., 2019). However, the steps may vary depending on the situation and the data quality. For example, in Hefron et al. (2018) the pre-processing consisted on trimming the trials, downsampling them to 512 Hz and 64 channels to reduce the complexity of the problem. Also, a high-pass filter was applied to the data, at the same time that the PREP (an standardized early-stage EEG processing) pipeline was used to calculate an average reference and remove line noise. On the other hand, the work carried in Stober et al. (2015) only applied a single pre-processing step of channel rejection. In the works done by Saha et al. (2019a,b) they used channel cross-covariance (CCV) for pre-processing; while in Cooney et al. (2019) they employed independent component analysis (ICA). Common average reference (CAR) method has also been employed to improve SNR from EEG signals by removing information that is present in all electrodes simultaneously (Moctezuma et al., 2019). Moreover, several studies have used temporal filtering as pre-process technique to focus on specific frequencies among the EEG signals (Jahangiri et al., 2018; Koizumi et al., 2018; Jahangiri and Sepulveda, 2019; Pawar and Dhage, 2020). Another preprocessing technique that has been applied is Laplacian filter (Zhao and Rudzicz, 2015), which is a spatial filter. However, this type of filters is not commonly used because it can lead to loss of important EEG information. In fact, most of pre-processing techniques can lead to loss of information, besides requiring an extra computational cost. Therefore, end-to-end learning methods that require minimum pre-processing are of currently of interest in EEG classification. However, classifying almost raw EEG signals is not an easy task and requires further study (Lee et al., 2020).

## 6. Feature Extraction Techniques in Literature

During feature extraction, the main objective is to obtain the most relevant and significant information that will aid to correctly classify the neural signals. This process can be carried on the time domain, frequency domain, and spatial domain. In the time domain, the feature extraction process is often done through statistical analysis, obtaining statistical features such as standard deviation (SD), root mean square (RMS), mean, variance, sum, maximum, minimum, Hjorth parameters, sample entropy, autoregressive (AR) coefficients, among others (Riaz et al., 2014; Iqbal et al., 2016; AlSaleh et al., 2018; Cooney et al., 2018; Paul et al., 2018; Lee et al., 2019). On the other hand, the most common methods used to extract features from the frequency domain include Mel Frequency Cepstral Coefficients (MFCC), Short-Time Fourier transform (STFT), Fast Fourier Transform (FFT), Wavelet Transform (WT), Discrete Wavelet Transform (DWT), and Continuous Wavelet Transform (CWT) (Riaz et al., 2014; Salinas, 2017; Cooney et al., 2018; Garćıa-Salinas et al., 2018; Panachakel et al., 2019; Pan et al., 2021). Additionally, there is a method called Bag-of-Features (BoF) proposed by Lin et al. (2012), in which a time-frequency analysis is done to convert the signal into words using Sumbolic Arregate approXimation (SAX). In the case of spatial domain analysis, the most common method used in several works is Common Spatial Patterns (CSP) (Brigham and Kumar, 2010; Riaz et al., 2014; Arjestan et al., 2016; AlSaleh et al., 2018; Lee et al., 2019; Panachakel et al., 2020). Moreover, it is important to mention that these feature extraction methods can be done in two different ways: from individual channels and simultaneously from multiple channels. Despite individual channel analysis being easier, extracting features from diverse channels at the same time is more useful because it helps to analyze how information is transferred between the different areas of the brain. In order to do a simultaneous feature extraction, the most common method is the channel cross-covariance (CCV) matrix; in which the features of each channel are fused together to enhance the statistical relationship between the different electrodes (Nguyen et al., 2017; Saha and Fels, 2019; Singh and Gumaste, 2021). In fact, Riemannian geometry is an advanced feature extraction technique that has been used to manipulate covariance matrices. It has been successfully applied toward several applications, such as motor imagery, sleep/respiratory states classification, EEG decoding, etc. (Barachant et al., 2010, 2011; Navarro-Sune et al., 2016; Yger et al., 2016; Chu et al., 2020).

## 7. Classification Techniques in Literature

In order to classify the features extracted from the EEG signal, researchers have used both classical machine learning and deep learning algorithms. Both of them are methods that provide computers the capacity of learning and recognizing patterns. In the case of BCI, the patterns to be recognized are the features extracted from the EEG waves, and then, based on what the computer learnt, some predictions are made in order to classify the signals. Several classical machine learning techniques have been used to approach imagined speech decoding for EEG-based BCI systems. Some on the most common algorithms include Linear Discriminant Analysis (LDA) (Chi et al., 2011; Song and Sepulveda, 2014; Lee et al., 2021b), Support Vector Machines (SVM) (DaSalla et al., 2009; Garćıa et al., 2012; Kim et al., 2013; Riaz et al., 2014; Sarmiento et al., 2014; Zhao and Rudzicz, 2015; Arjestan et al., 2016; González-Castañeda et al., 2017; Hashim et al., 2017; Cooney et al., 2018; Moctezuma and Molinas, 2018; Agarwal and Kumar, 2021), Random Forests (RF) (González-Castañeda et al., 2017; Moctezuma and Molinas, 2018; Moctezuma et al., 2019), k-Nearest-Neighbors (kNN) (Riaz et al., 2014; Bakhshali et al., 2020; Agarwal and Kumar, 2021; Rao, 2021; Dash et al., 2022), Naive Bayes (Dash et al., 2020a; Agarwal and Kumar, 2021; Iliopoulos and Papasotiriou, 2021; Lee et al., 2021b), and Relevance Vector Machines (RVM) (Liang et al., 2006; Matsumoto and Hori, 2014). Furthermore, deep learning approaches have recently taken a huge role for imagined speech recognition. Some of these techniques are Deep Neural Networks (DBN) (Lee and Sim, 2015; Chengaiyan et al., 2020), Correlation Networks (CorrNet) (Sharon and Murthy, 2020), Standardization-Refinement Domain Adaptation (SRDA) (Jiménez-Guarneros and Gómez-Gil, 2021), Extreme Learning Machine (ELM) (Pawar and Dhage, 2020), Convolutional Neural Networks (CNN) (Cooney et al., 2019, 2020; Tamm et al., 2020), Recurrent Neural Networks (RNN) (Chengaiyan et al., 2020), and parallel CNN+RNN with and without autoencoders autoencoders (Saha and Fels, 2019; Saha et al., 2019a,b; Kumar and Scheme, 2021).

## 8. Discussion, Applications, and Limitations of Previous Research

Based on the previous sections and the diverse works mentioned in them, imagined speech classification can be summed up as in Tables 1–6.

**Table 1:** Imagined speech classification methods summary.

**References**	**Task**	**Methods**	**Brain area and waves**	**Performance**
D'Zmura et al. (2009)	Binary classification /ba/ and /ku/ syllables	- Hilbert transform	- All with exception of 18 electrodes most sensitive to electromyographic artifact	61% Average accuracy
		- Matched filters	-Alpha, beta, and theta waves	
DaSalla et al. (2009)	Binary classification between /a/, /u/ and rest state	- CSP	- All areas	71% Average accuracy
		- SVM	− 1–45 Hz bandpass	
Brigham and Kumar (2010)	Binary classification /ba/ and /ku/ syllables	- ICA	- All with exception of 18 electrodes most sensitive to electromyographic artifact	68% Average accuracy
		- AR model	− 4–25 Hz bandpass	
		- KNN		
Deng et al. (2010)	Binary classification /ba/ and /ku/ syllables	- SOBI algorithm	- All areas	67% Average accuracy
		- Hilbert spectrum	− 3–20 Hz bandpass	
		- FFT / STFT		
		- LDA		
Chi et al. (2011)	Binary classification of five phoneme classes	- Naive Bayes	- All areas omitting occipital and far frontal positions	72% Average accuracy
		- LDA	− 4–28 Hz bandpass	
		- Spectrogram		

**Table 2:** Imagined speech classification methods summary (continuation).

**References**	**Task**	**Methods**	**Brain area and waves**	**Performance**
Garćıa et al. (2012)	Multi-class classification of five words	- Naive Bayes	- Wernicke's area	26% Average accuracy
		- SVM	- -25 Hz bandpass	
		- Random Forests		
Matsumoto and Hori (2014)	Binary classification of /a/, /e/, /i/, /o/, and /u/ Japanese vowels	- CSP	- All areas	−79% RVM Average accuracy
		- RVM	−0.1–300 Hz bandpass	−77% SVM Average accuracy
		- SVM		
Sarmiento et al. (2014)	Binary classification of /a/, /e/, /i/, /o/, and /u/	- ANOVA	- Broca's area and Wernicke's area	−79% RVM Average accuracy
		- Power spectral density	−2–50 Hz bandpass	
		- SVM		
Riaz et al. (2014)	Binary classification of /a/, /e/, /i/, /o/, and /u/	- AR coefficients	- All areas	75% Average accuracy
		- Hidden Markov Model	-Alpha and beta waves	
		- KNN / SVM		
		- CSP / MFCC / LDA		
Zhao and Rudzicz (2015)	Binary classification for presence of C/V, ± Nasal, ± Bilab,± /uw/, ± /iy/	- DBN	- T7, FT8, FC6, C5, C3, CP3, C4, CP5, CP1, P3	C/V: 18% Accuracy
		- SVM	−1–50 Hz bandpass	± Nasal: 63.5% Accuracy
				± Bilab: 56.6% Accuracy
				± /uw/: 59.6% Accuracy
				± /iy/: 79.1% Accuracy

**Table 3:** Imagined speech classification methods summary (continuation).

**References**	**Task**	**Methods**	**Brain area and waves**	**Performance**
Arjestan et al. (2016)	Binary classification vowels, syllables, and resting state	- CSP	- All areas	Vowels: 76.6% best accuracy
		- SVM	− 8–45 Hz bandpass	Syllables: 76.4% best accuracy
Sereshkeh et al. (2017)	Binary classification of “yes,” “no,” and rest state	- Multilayer perceptron	- All areas	70% Average accuracy
		- DWT	− 0–50 Hz bandpass	
		- RMS / SD		
Nguyen et al. (2017)	Classification of vowels, short, and long words	- Riemannian Manifold	- Broca's area, the motor cortex and Wernicke's area	- Vowels: 49% Average accuracy
		- RVM	− 8–70 Hz bandpass	- Short words: 50.1% Average accuracy
		- CSP / WT		- Long words: 66.2% Average accuracy
				- S-L: 80.1% Average accuracy
Paul et al. (2018)	Classification of three Hindi vowels	- SVM	- Broca's and Wernicke's area	63% Average accuracy
		- AR coefficients	− 0.1–36 Hz bandpass	
		- Hjorth parameters		
		- Sample entropy		
Cooney et al. (2018)	Classification of seven phonemic prompts and four words	- SVM	- All areas	- Phonemes: 20% Average accuracy
		- Decision Tree	− 1–50 Hz bandpass	- Pair of words: 44% Average accuracy
		- MFCC		
		- ICA		

**Table 4:** Imagined speech classification methods summary (continuation).

**References**	**Task**	**Methods**	**Brain area and waves**	**Performance**
Moctezuma and Molinas (2018)	Classification of “up,” “down,” “right,” “left,” “select”	- RF	- All areas	- RF: 64% Average accuracy
		- SVM	−1–50 Hz bandpass	- SVM: 84% Average accuracy
		- Naive Bayes		- Naive Bayes: 68% Average accuracy
		- KNN		- KNN: 78% Average accuracy
		- EMD/IMF		
Saha and Fels (2019)	Classification of vowels, short and long words	- CCV	- All areas	- Vowels: 72% Average accuracy
		- CNN + LSTM + DAE		- Short words: 77% Average accuracy
				- Long words: 79% Average accuracy
Saha et al. (2019a)	Binary classification for presence of C/V, ± Nasal, ± Bilab,± /uw/, ± /iy/	- CCV	- All areas	- C/V: 85% Accuracy
		- CNN + TCNN + DAE		± Nasal: 73% Accuracy
				± Bilab: 75% Accuracy
				± /uw/: 82% Accuracy
				± /iy/: 73% Accuracy
				± Multiclass: 28% Accuracy
Panachakel et al. (2019)	Classification of seven phonemic prompts and four words	- DNN	- C4, FC3, FC1, F5, C3, F7, FT7, CZ, P3, T7, C5	−57% Average accuracy
		- DWT	−1–50 Hz bandpass	
Moctezuma et al. (2019)	Classification of “up,” “down,” “right,” “left,” “select”	- RF	- All areas	−93% Average accuracy
		- CAR	−0–64 Hz bandpass	
		- DWT / Statistics		

**Table 5:** Imagined speech classification methods summary (continuation).

**References**	**Task**	**Methods**	**Brain area and waves**	**Performance**
Cooney et al. (2019)	Classification of /a/, /e/, /i/, /o/, and /u/	- CNN	- All areas	−34% Average accuracy
		- Transfer learning	−2–40 Hz bandpass	
Sharon and Murthy (2020)	Binary classification for presence of C/V, ± Nasal, ± Bilab,± /uw/, ± /iy/	- CorrNet	- All areas	C/V: 89% Accuracy
			−1-50 Hz bandpass	± Nasal: 76% Accuracy
				± Bilab: 75% Accuracy
				± /uw/: 82% Accuracy
				± /iy/: 80% Accuracy
Bakhshali et al. (2020)	Binary classification for presence of C/V, ± Nasal, ± Bilab,± /uw/, ± /iy/	- Riemannian distance	- Broca's area and Wernicke's area	C/V: 86% Accuracy
	Binary Classification of /pat/, /pot/, /gnaw/, and /knew/	- Correntropy Spectral Density	−1–50 Hz bandpass	± Nasal: 72% Accuracy
				± Bilab: 69% Accuracy
				± /uw/: 84% Accuracy
				± /iy/: 75% Accuracy
				± Word binary classification: 69% Average accuracy
Pawar and Dhage (2020)	Muti-class and binary classification of “left,” “right,” “up,” and “down”	- Kernel ELM	- All areas	Multi-class: 49% best accuracy
		- Statistical features	- Prefrontal cortex, Wernicke's area, right inferior frontal gyrus, Broca's area	Binary: 85% best accuracy
		- DWT	- Prefrontal cortex, Wernicke's area, right inferior frontal gyrus, Broca's area, primary motor cortex	
		- ICA	−0.5–128 Hz bandpass	

**Table 6:** Imagined speech classification methods summary (continuation).

**References**	**Task**	**Methods**	**Brain area and waves**	**Performance**
Cooney et al. (2020)	- Classification of /a/, /e/, /i/, /o/, and /u/	- CNN	- All areas	- Vowels: 35% best accuracy
	- Classification of “left,” “right,” “up,” “down,” “forward,” “backward” (in Spanish)	- ICA/LDA	−2–40 Hz bandpass	- Words: 30% best accuracy
Tamm et al. (2020)	Classification of five vowels and six words	- CNN	- F3, F4, C3, C4, P3, P4	−24% Average accuracy
		- Transfer learning		
Chengaiyan et al. (2020)	Vowel classification	- RNN	- All areas	- RNN: 72% Average accuracy
		- DBN	−2–40 Hz bandpass	- DBN: 80% Average accuracy
Jiménez-Guarneros and Gómez-Gil (2021)	Short and long words classification	- SRDA	- All areas	- Short: 61% Average accuracy
				- Long: 63% Average accuracy

As observed in the previous tables, there have been different attempts to achieve a good performance of imagined speech recognition using EEG-based BCI. These attempts involve diverse feature extraction and classification methods. Therefore, in Tables 7, 8 we offer a summary of the advantages and disadvantages of some of these methods.

**Table 7:** Comparison of feature extraction methods.

**Method**	**Advantages**	**Disadvantages**
AR	- Good frequency resolution	- Low performance when applied to non-stationary signals
	- Limited spectral loss	- The order of the model is difficult to select
FFT	- Good performance when applied to stationary signals	- High noise sensitivity
	- Appropriate for narrowband signals	- Poor performance on non-stationary signals
	- Good speed for real-time applications	- Weak spectral estimation
WT	- Varying window size to analyze several frequencies	- Proper mother wavelet selection is not trivial
	- Good to analyze transient signal changes	

**Table 8:** Comparison of classification methods.

**Method**	**Advantages**	**Disadvantages**
KNN	- Easy to understand and implement	- Large storage capacity is needed
		- Error susceptibility and sensitivity to irrelevant features
SVM	- Effective in high dimensional spaces	- Poor performance on noisy and large datasets
	- Relatively low storage capacity is needed	
LDA	- Simple to understand and use	- Requires a linear model
ANN	- Relatively high accuracy	- Requires large datasets for it to be trained
	- Flexible and adaptable structure	- High computational cost
	- Handles multidimensional data	- Performance depends on several parameters, such as number of neurons and hidden layers
DL algorithms	- Robustness for adaptation	- Requires large datasets for it to perform well
	- In can be adapted to different problems through transfer learning	- Need high computational resources
	- Features can be automatically deduced and tuned	- Difficult to implement for novices

The main objective of most imagined speech decoding BCI is to provide a new communication channel for those who have partial or total movement impairment (Rezazadeh Sereshkeh et al., 2019). Nevertheless, besides speech restoration, there are some other novel applications of imagined speech decoding that have been explored. In Kim et al. (2020), researchers proposed a BCI paradigm that combined event-related potentials and imagined speech to target individual objects in a smart home environment. This was done through EEG analysis and classification using regularized linear discriminant analysis (RLDA). Moreover, the work presented in Asghari Bejestani et al. (2022) focused on the classification of six Persian words through imagined speech decoding. These words, as said by the authors, can be used to control electronic devices such as a wheelchair or to fill a simple questionnaire form. Tøttrup et al. (2019) explored the possibility of combining motor imagery and imagined speech recognition for controlling an external device through EEG-based BCI and random forest algorithm. Furthermore, the work presented by Moctezuma and Molinas (2018) explored the application of imagined speech decoding toward subject identification using SVM.

Regardless of the rising interest on EEG-based BCI for imagined speech recognition, the development of systems that are useful for real-life applications is still in its infancy. In the case of syllables, vowels, and phonemes, the limited amount of vocabulary that has been analyzed impedes the possibility of applying BCI to allow people to speak through their thoughts. Among all the reviewed proposals, the one that seems closer to be applied in real life is the classification of words such as “up,” “down,” “left,” “right,” “forward,” “backward,” and “select.” The reason behind this is that those words can be used to control external devices such as a computer/cellphone screen and robotic prosthesis. However, the fact of those words being classified by EEG-based BCI systems that are offline and synchronous makes the projects less scalable to real-life applications.

Also, it is important to mention that EEG-based BCI lacks from accuracy when compared with other methods such as ECoG and MEG. ECoG has been applied in several studies for either covert and overt speech decoding, achieving higher average accuracies than EEG-based BCI. For example, in Martin et al. (2016) imagined speech pairwise classification reached an accuracy of 88.3% through ECoG recording. Kanas et al. (2014) presented a spatio-spectral feature clustering of ECoG recordings for syllable classification, obtaining an accuracy of 98.8%. Also, a work performed by Zhang et al. (2012) obtained a 77.5% accuracy on the classification of eight-character Chinese spoken sentences through the analysis of ECoG recordings. Moreover, in the work presented by Dash et al. (2019) MEG was used for phrase classification, achieving a top accuracy of 95%. Finally, the study in Dash et al. (2020a) aimed to classify articulated and imagined speech on healthy and amyotrophic lateral sclerosis (ALS) patients. In this work the best articulation decoding accuracy for ALS patients was 87.78%, while for imagined decoding was 74.57%.

In summary, the past research allowed to observe the following current limitations of EEG-based BCI systems for imagined speech recognition:

- Limited vocabulary: Most of the reviewed studies focused on imagined vowels (/a/, /e/, /i/, /o/, /u/, /ba/, /ku/) and words such as “right,” “left,” “up,” and “down.” This shows how far away we are from truly decode enough vocabulary for a real-life application of covert speech decoding.
- Limited accuracy: Despite some works reaching +80% accuracy, this was achieved mostly for binary classification. Multi-class classification, which would be more viable for real-life application, demonstrated to have much lower classification rates than binary tasks. It is important to notice that even binary accuracy decreases or increases depending on the nature of the task to be done (for example: long vs. short words compared to words of the same length).
- Mental repetition of the prompt: The experimental design of most studies included the repeated imagination of the vowel, phoneme or word. This helps increasing the accuracy of the algorithm; however, mental repetition is not included on daily conversation tasks. Therefore, the design of some proposed experiments have low reliability when considering their practical application.
- Acquisition system: Most of the reviewed works used a high-density EEG system, which may be difficult to apply in real-life situations Also, almost no work reviewed in here deals with an online and asynchronous BCI system, which, as mentioned earlier, is the feasible BCI option for practical applications.

## 9. Conclusions and Future Work

The rapid development of the Future Internet framework has led to several new applications such as smart environments, autonomous monitoring of medical health, cloud computing, etc. (Zhang et al., 2019). Moreover, there are important future plans, such as Internet Plus and Industry 4.0, that require further integration of internet with other areas, such as medicine and economics. Therefore, technologies such as Brain Computer Interfaces seem to be promising areas to be explored and implemented to solve real-life problems.

Through this review, we analyzed works that involved EEG-based BCI systems directed toward imagined speech recognition. These works followed the decoding of imagined syllables, phonemes, vowels, and words. However, the study of each of those groups was individual, meaning that there was no work aiming to study vowels vs. words, phonemes vs. words, phonemes vs. vowels, etc. at the same time. Also, it is important to notice that each BCI was used for a single person, which would make difficult the implementation of a general and globalized system. It seems that each individual would need to train their own BCI system in order to use it successfully.

Another thing to take into account is that several languages have been analyzed, such as English, Spanish, Chinese, and Hindi. However, there is not a comprehensive study that evaluates the impact of how a method performs toward an specific language.

Regarding feature extraction methods, there have been a large amount of proposed techniques such as DWT, MFCC, STFT, CSP, Riemannian space, etc. On the other hand, the most studied classification algorithm has been SVM, which is a classical machine learning technique. Deep learning techniques such as CNN and RNN have also been explored by some authors. Despite deep learning showing promising accuracy improvements in comparison to classical ML, it is difficult to fully exploit it because of the limited amount of data available to train DL algorithms.

Additionally, currently there is not definitive information regarding the most important EEG recording locations of imagined speech recognition. Broca's and Wernicke's areas are well-known to be involved in speech production; however, some studies reviewed here showed that they are not the only zones that contain valuable information for covert speech decoding. Therefore, it seems a good idea to propose a method that helps selecting the EEG channels that better characterize a given task.

All things considered, we identified the following tasks as promising for the future development of EEG-based BCI systems for imagined speech decoding:

- Broaden the existing datasets in such a way that deep learning techniques could be applied to their full extent. Moreover, explore and propose prompts that could be more easily applied to solve real-life problems.
- Find and propose more varied prompts in order to enhance the difference between their EEG signatures and detect the most discriminative characteristic to enhance classification. This can be done by employing different rhythms, tones, overall structure, and language.
- Explore how a same proposed method performs over different languages.
- Recognize the best feature extraction and machine learning techniques to improve classification accuracy. At the same time, there is still room for improvement in the identification of EEG frequency range that offers the most valuable information.
- Most of the current studies are offline-synchronous BCI systems applied in healthy subjects. Also, most experiments are highly controlled in order to avoid artifacts. Therefore, there is room for further work in these areas.
- Explore different imagery processes, such as Visual Imagery (Ullah and Halim, 2021).

## Author Contributions

DL-B: formal analysis, investigation, methodology, and writing—original draft. PP and AM: resources. DB, PP, and AM: supervision, validation, and writing—review and editing. All authors have read and agreed to the published version of the manuscript.

## Funding

This work was funded by Fondo para el financiamiento para la publicación de Artículos Cientof Monterrey Institute of Technology and Higher Education.

## Conflict of Interest

The authors declare that the research was conducted in the absence of any commercial or financial relationships that could be construed as a potential conflict of interest.

## Publisher's Note

All claims expressed in this article are solely those of the authors and do not necessarily represent those of their affiliated organizations, or those of the publisher, the editors and the reviewers. Any product that may be evaluated in this article, or claim that may be made by its manufacturer, is not guaranteed or endorsed by the publisher.
